# Alterations in soil microbial community composition and biomass following agricultural land use change

**DOI:** 10.1038/srep36587

**Published:** 2016-11-04

**Authors:** Qian Zhang, Junjun Wu, Fan Yang, Yao Lei, Quanfa Zhang, Xiaoli Cheng

**Affiliations:** 1 Key Laboratory of Aquatic Botany and Watershed Ecology, Wuhan Botanical Garden, Chinese Academy of Sciences, Wuhan 430074, P. R. China; 2 Graduate University of Chinese Academy of Sciences, Beijing, 10039, China

## Abstract

The effect of agricultural land use change on soil microbial community composition and biomass remains a widely debated topic. Here, we investigated soil microbial community composition and biomass [e.g., bacteria (B), fungi (F), Arbuscular mycorrhizal fungi (AMF) and Actinomycete (ACT)] using phospholipid fatty acids (PLFAs) analysis, and basal microbial respiration in afforested, cropland and adjacent uncultivated soils in central China. We also investigated soil organic carbon and nitrogen (SOC and SON), labile carbon and nitrogen (LC and LN), recalcitrant carbon and nitrogen (RC and RN), pH, moisture, and temperature. Afforestation averaged higher microbial PLFA biomass compared with cropland and uncultivated soils with higher values in top soils than deep soils. The microbial PLFA biomass was strongly correlated with SON and LC. Higher SOC, SON, LC, LN, moisture and lower pH in afforested soils could be explained approximately 87.3% of total variation of higher total PLFAs. Afforestation also enhanced the F: B ratios compared with cropland. The basal microbial respiration was higher while the basal microbial respiration on a per-unit-PLFA basis was lower in afforested land than adjacent cropland and uncultivated land, suggesting afforestation may increase soil C utilization efficiency and decrease respiration loss in afforested soils.

Land use change is a key component of global changes and largely impacts ecosystem structures, processes and functioning^1^,^2^,^3^. While agricultural production systems have been considered to be the primary cause of rapid carbon (C) loss^4^,^5^,^6^, forest regeneration or reforestation (i.e., afforestation) conducted on formerly cultivated or uncultivated lands can sequester C in aboveground biomass and in soil organic matter (SOM)^7^,^8^,^9^. Reforestation or afforestation is an approach to restore forests that reduces the effects of climate change^7^. Soil microorganisms are the decomposers of litter and SOM in terrestrial ecosystems, which can regulate multiple input and loss pathways of soil C and nitrogen (N)^10^,^11^. Changes in microbial community structure and function are hypothesized to alter ecosystem processes, such as plant litter decomposition, and nutrient availability^10^,^12^. It has been suggested that land use change can affect the microbial decomposition of litter and SOM, which in turn regulates soil C and N balance in terrestrial ecosystems^12^,^13^. Thus, evaluating the effects of land use change on the soil microbial community structure is important for better understanding human effects on the global C cycle.

Shifts in plant species composition during agricultural land use change can impact microbial community structure and biomass primarily by altering soil organic C and N input^14^,^15^. For instance, previous studies have found that afforestation usually increases soil C and N inputs and then stimulates microbial activities as they are sources of nutrients and energy to microorganisms^16^. Whereas the different chemical compositions of the plant residues and SOM following land use change have great effects on microbial activity and microbial community structure^17^,^18^, different microbial groups usually use different sources and amounts of C^19^,^20^. For instance, Urbanová *et al*.^21^ have reported that the effects of the tree species in a forest ecosystem explain a large proportion of variation in microbial community composition than other soil properties, especially in fungi^21^. Meanwhile, the G^−^ bacteria are found to prefer recent plant-derived carbon and G^+^ bacteria are found to prefer older SOM-derived carbon^19^. The relative abundance of fungi to bacteria (i.e., F: B ratios) is usually sensitive to soil disturbance with lower ratios associated with higher frequencies of tillage^22^,^23^. Some studies have proposed that the conventional tillage involved in agricultural practices can result in a more bacterial-dominated system instead of a fungal-dominated system compared with no-till agricultural practices^22^,^24^. Changes in substrate quality can also alter F: B ratios, because substrate with low C: N ratio usually favors bacteria and it with high C: N ratio usually favors fungi^25^. Thus, it is expected that agricultural land use change may impact soil microbial community composition and biomass, but more convincing data are still in warranted.

Microbial community composition and biomass can be impacted by land use change primarily by soil properties such as pH, soil depth, moisture and temperature^26^,^27^,^28^. Soil pH can greatly affect the F: B ratios^29^,^30^, especially the relative concentration and diversity of bacteria^31^. Some studies have noted that fungi are more acid tolerant than bacteria resulting increased fungal dominance in acid soils^30^,^32^. Meanwhile, Stevenson (2014) has reported that soil moisture is very important for all microbial communities^33^, generally, fungi will be less sensitive to changes in moisture than bacteria because their chitinous cell walls make them more resilient to changes in moisture and temperature^34^. In addition, the abundance, composition, and biomass of microbial communities within soils are strongly dependent with the increase in soil depth due to different C availability^29^,^35^.

In recent decades, afforestation (implementing woodland and shrubland plantations) has been conducted on formerly cultivated or uncultivated lands to protect water quality and restore riparian ecosystem function in the Danjiangkou Reservoir of central China, which is a water source for the central route of the China South-to-North Water Transfer Project^36^. Previous studies have reported that afforestation in this region could enhance soil C sequestration due to increases in litter inputs^37^. Shifts of agriculture land to shrubland and woodland also can reduce soil net N mineralization^38^ and soil erosion^39^. However, there is still a lack of information about how the soil microbial community reacts to land use change in this area. In this study, we hypothesized that land use change would significantly alter microbial community and biomass and the basal microbial respiration due to alterations in soil C and N availability and soil physiochemical properties. To test these hypothesis, we investigated the soil microbial community composition and biomass [e.g., bacteria (B), fungi (F), Arbuscular mycorrhizal fungi (AMF) and Actinomycete (ACT)] derived from phospholipid fatty acids (PLFAs) and basal microbial respiration from 30 days incubations of the top (0–10 cm) and deep soil (10–30 cm) in afforested land (implementing woodland and shrubland plantations) compared with adjacent cropland and uncultivated fields. We also examined soil chemical and physical properties including pH, moisture, temperature and soil organic carbon and nitrogen (SOC and SON), labile carbon and nitrogen (LC and LN) and recalcitrant carbon and recalcitrant nitrogen (RC and RN).

## Results

### Soil physicochemical characteristics

Soil moisture was highest in the woodland followed by cropland, shrubland and uncultivated land, whereas soil temperature showed the opposite tendency with the highest values in uncultivated land. The soil pH values were lower in the afforested soils than in the cropland and uncultivated soils. The SOC, SON, LC, LN, RC and RN levels were generally higher in the afforested soils than in the cropland and uncultivated soils, with higher values in top soil (0–10 cm) than deep soil (10–30 cm) (Table 1).

### Soil microbial community composition and biomass

**Figure 1:**
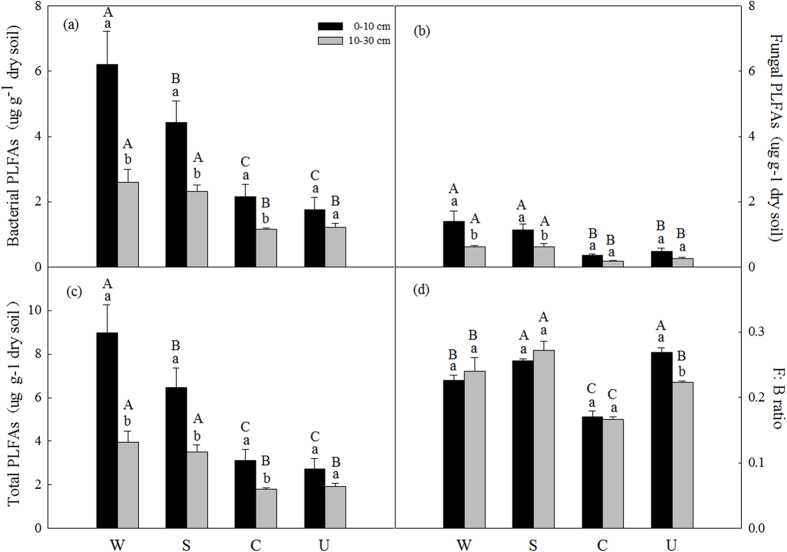
Soil total bacteria PLFAs (**a**), total Fungi PLFAs (**b**), total PLFAs (**c**) and F: B ratios (**d**) at different soil depths (0–10 cm and 10–30 cm) under different land use types. Values are Mean ± SE (n = 9). Values followed by a different lowercase letter are significant difference between 0–10 cm and 10–30 cm under same land use types. Values followed by a different capital letter are significant difference among land use types under same soil depth. Abbreviations: W, woodland; S, shrubland; C, cropland; U, uncultivated land.

The bacterial PLFAs and fungal PLFAs were significantly higher in afforested (woodland and shrubland) soils than in cropland and uncultivated land soils at both two soil layers (Fig. 1a,b). Total PLFAs were significantly higher in woodland and shrubland soils compared to the cropland soils and uncultivated land soils at both soil layers (Fig. 1c). The F: B ratios were significantly higher in afforested land soils than cropland soils (Fig. 1d). There was no significant difference in F:B ratios between uncultivated land soils and shrubland soils at top soil layers and woodland soils at deeper soil layers (Fig. 1d). The bacterial PLFAs and total PLFAs were significantly higher in top soil than deep soil (except in uncultivated land soils) and the fungal PLFAs were significantly higher in top soil than deep soil in afforested land soils (Table 2; Fig. 1a,b), while there were no significant differences in F: B ratios between two soil layers in all land use types except uncultivated land soils (Table 2; Fig. 1d).

**Figure 2:**
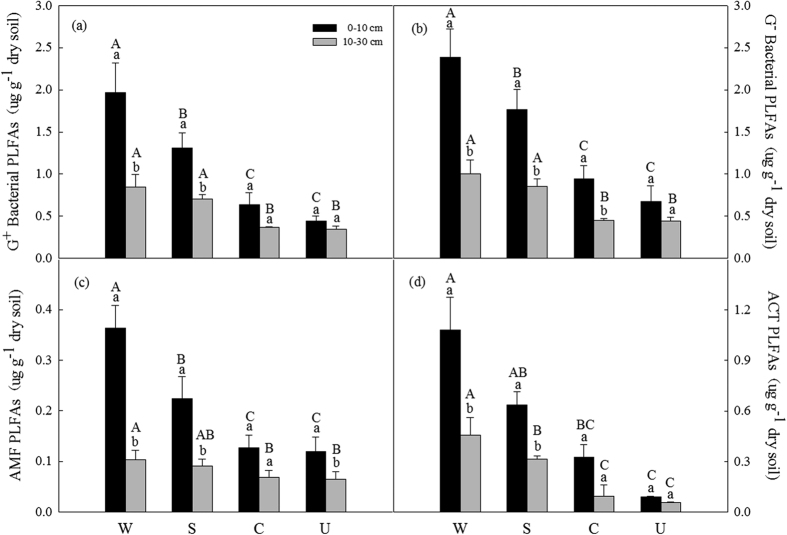
Soil G^+^ bacteria PLFAs (**a**), G^−^ bacteria PLFAs (**b**), AMF PLFAs (**c**) and ACT PLFAs (**d**) at different soil depth (0–10 cm and 10–30 cm) under different land use types. Values are Mean ± SE (n = 9). Values followed by a different lowercase letter are significant difference between 0–10 cm and 10–30 cm under same land use types. Values followed by a different capital letter are significant difference among land use types under same soil depth. See Fig. 1 for the abbreviations.

The gram-positive bacterial (G^+^ bacterial) PLFAs, gram-negative bacterial (G^−^ bacterial) PLFAs, Arbuscular mycorrhizal fungi (AMF) PLFAs and Actinomycete (ACT) PLFAs displayed similar pattern with higher levels in the afforested soils than cropland and uncultivated land soils at both soil layers (Fig. 2). All the G^+^ bacterial PLFAs, G^−^ bacterial PLFAs, AMF PLFAs and ACT PLFAs were significantly higher in top soil layers than in deeper soil layers except in uncultivated land soils (Table 2; Fig. 2).

### Soil basal microbial respiration

**Figure 3:**
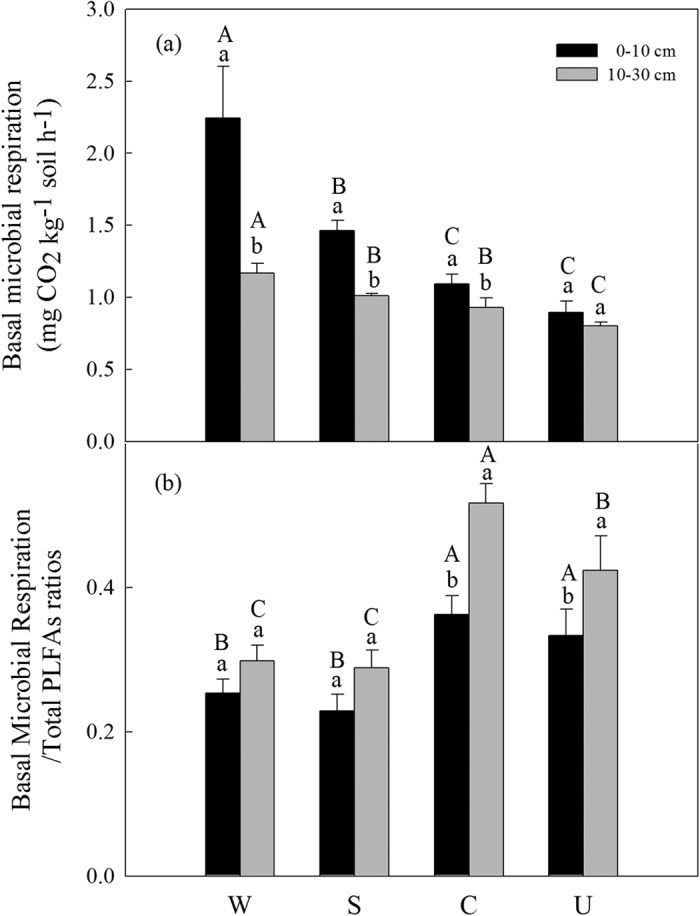
Basal microbial respiration (**a**), and basal microbial respiration on a per-unit-PLFA basis (**b**) at different soil depths (0–10 cm and 10–30 cm) under different land use types. Values are Mean ± SE (n = 9). Values followed by a different lowercase letter are significant difference between 0–10 cm and 10–30 cm under same land use types. Values followed by a different capital letter are significant difference among land use types under same soil depth. See Fig. 1 for the abbreviations.

The basal microbial respiration of top soil significantly increased from cropland to shrubland to woodland with no significant difference between cropland and uncultivated land (Fig. 3a), while the basal microbial respiration of deep soil was significantly higher in woodland and significantly lower in uncultivated land than other land use types with no significant difference between shrubland and cropland (Fig. 3a). The ratios of the basal microbial respiration to total PLFA biomass were significantly lower in the afforested soils than in the cropland and uncultivated soils at both soil layers (Fig. 3b). Soil basal microbial respiration decreased with increasing soil depth except uncultivated soils (Fig. 3a), and the ratio of the basal microbial respiration to the total PLFAs increased with increasing soil depth in cropland and uncultivated soils (Fig. 3b).

### Relationships between soil microbial community composition and environmental factors

**Figure 4:**
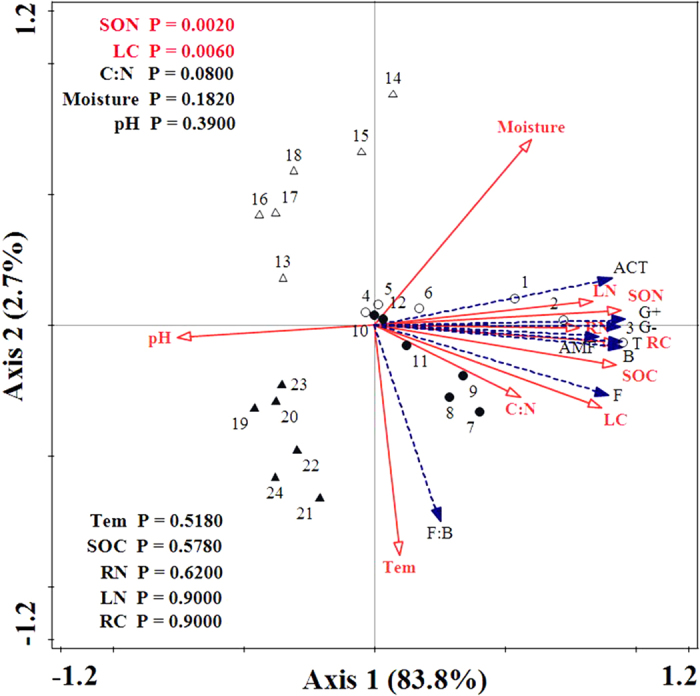
Redundancy analysis (RDA) results of PLFAs profiles for the soil samples used seven PLFAs as species and ten environmental variables. The variables are shown by different arrows: PLFAs profiles (species) by blue arrows: total PLFAs (T); bacterial PLFAs (B); fungi PLFAs (F); Gram-positive bacterial PLFAs (G+); Gram-negative bacterial PLFAs (G−); arbuscular mycorrhizal fungal PLFAs (AMF); actinomycete PLFAs (ACT); and environmental variables by the red dashed arrow: soil temperature (Tem); soil moisture; soil pH; soil organic carbon (SOC); soil organic nitrogen (SON); ratio of SOC to SON (C: N); soil labile organic carbon (LC); soil labile organic nitrogen (LN); soil recalcitrant carbon (RC) and soil recalcitrant nitrogen (RN). Empty circles represent woodland, filled cycles represent shrubland, empty up-triangles represent cropland and filled up-triangles represent uncultivated land. Numbers on the plot mean P-values.

There was a significant relationship between the composition of soil microbial community and environment factors (Fig. 4; F = 14.1, P = 0.002). All ten environmental factors (i.e., soil moisture, soil temperature, pH, SOC, SON, C: N, RC, RN, LC and LN) explained 86.5% of the total variability in the PLFAs (Fig. 4). The results of Monte Carlo permutation tests (P < 0.05) showed that the variability of the PLFAs was strongly related to SON (F = 129.49, P = 0.002) and LC (F = 9.41, P = 0.006) (Fig. 4). The first ordination RDA axis (axis 1, horizontal), which was strongly related with SON and LC, explained 83.8% of the variability in the PLFAs (Fig. 4). The second ordination RDA axis (axis 2, vertical) was mainly related to temperature and explained 2.7% of the total variability in the PLFAs (Fig. 4). The bacterial PLFAs, fungal PLFAs, G^+^ bacterial PLFAs, G^−^ bacterial PLFAs, ACT PLFAs, AMF PLFAs and total PLFAs were positively correlated with moisture, SOC, SON, C: N ratio, LC, LN, RC, RN and temperature, and negatively correlated with pH (Fig. 4). The Pearson correlation analysis showed that all of the PLFAs were significantly positively correlated with moisture, SOC, SON, LC, LN, RC, RN (Table 3). The total PLFAs, bacterial PLFAs, fungal PLFAs, G^+^ bacterial PLFAs and ACT PLFAs were significantly related to C: N ratio (Table 3). The F: B ratios were significantly positively correlated with soil temperature, pH, the C: N ratios and LC, but negatively related to the soil moisture across land use types (Table 3). The basal microbial respiration was significantly related to soil moisture, SOC, SON, LC, LN, RC, and RN, but negatively related to soil pH. The basal microbial respiration to total PLFA biomass ratios were positively related to soil pH, soil C: N ratio and LC while negatively related to soil moisture.

## Discussion

This study demonstrated that land use change greatly impacted microbial community and biomass, as well as the basal microbial respiration in the Danjiangkou Reservoir area of central China (Figs 1, 2, 3 and 4). Vegetation types and belowground components are strongly linked through a variety of direct and indirect interactions on microbial activities^14^,^40^. In the present study, we found that afforestation averaged higher microbial PLFA biomass compared with cropland and uncultivated land soils at both soil layers (Fig. 1c). Soil C and N availabilities have been considered as key driving factors for microbial community dynamics^41^. Increases in all types of microbial PLFA biomass (Figs 1 and 2) are possibly attributed to increased litter input and soil organic C and N content in the afforested soils^37^,^38^ (Table 1). This speculation was supported by our Pearson’s correlation analysis and redundancy analysis that the quantities of total PLFAs and various microbial types of PLFAs were strongly dependent on the soil C and N availability (Table 3; Fig. 4). Additionally, all types of PLFAs decreased with the soil depth across all land use types (Table 2; Figs 1, 2, 3 and 4), which was mainly attributed to the decrease in soil C and N availability with increasing soil depth^5^,^37^ (Table 1).

Nevertheless, differences in microbial community composition following land use change also have been attributed to differences in soil properties^13^,^15^,^42^. For example, land use change could lead to changes in soil pH, soil moisture and temperature^43^,^44^, which in turn would impact microbial biomass and activity as soil microbial organisms could respond quickly to changes in soil environment^2^. The key role of soil properties in regulating the microbial community was clear in this study, as indicated by the evidence that the total PLFAs and various microbial types of PLFAs were significantly positively related to soil moisture and negatively related to soil pH (Table 3).

The fungal: bacterial PLFAs (F: B) ratio has been proposed to evaluate the responses of the soil microbial community to soil C and N dynamics and environmental changes^23^,^45^. Bossuyt *et al*.^25^ have also reported that the activity of bacteria is more sensitive to low availability of C and N than fungi, while fungi prefer low quality substrates (high C: N)^25^. Increases in the F: B ratios in the afforested land compared with cropland (Fig. 1) can be due to more low-quality litter input because bacteria require more N per unit biomass C accumulation than fungi^29^. Indeed, we found that the F: B ratios were significantly positively related to soil C: N ratios across land use types and soil depth (Table 3; Fig. 4). Meanwhile, fungi were found to be more resistant to acid than bacteria^30^,^32^, and the lower pH in afforested soil (Table 1) possibly led to higher F: B ratios (Table 1; Fig. 1d). Additionally, the relative higher fungal activitiy was found under warmer and drier environments and bacteria activity under colder and moister environments^46^,^47^,^48^. Low soil temperature and high moisture in the afforested land (Table 1; Table 3) likely resulted in greater F: B ratios in the afforested soil. Moreover, bacteria can be relatively unaffected by tilling compared with fungi^22^,^24^ and afforestation of cropland could enhance the F: B ratios given decrease tillage^33^.

Afforestation also enhanced soil Gram-negative bacteria (G^−^ bacteria) biomass, Gram-positive bacteria (G^+^ bacterial) biomass, Actinomycete (ACT) and Arbuscular mycorrhizal fungi (AMF) biomass (Fig. 2), with higher G^−^ bacterial biomass than G^+^ bacterial biomass across all land use types (Fig. 2a,b). G^−^ bacteria has been thought to prefer high carbon availability^9^,^19^, greater G^−^ bacterial biomass relative to G^+^ bacterial biomass in all land use types may mean a high copiotrophic condition in our study area^49^. Both G^−^ bacterial land G^+^ bacterial PLFA biomass could be affected by soil pH^50^, our results showed the negative relationship of G^−^ bacterial and G^+^ bacterial PLFA biomass with pH (Table 3). Higher intensity levels of agricultural management tend to produce a lower AMF richness^51^. Actinomycetes are major decomposers of complex polymers in soil and are affected by the tillage regime^52^. AMF and ACT biomass were more abundant in afforested soils compared with in cropland (Fig. 2c,d), which might be associated with high SOC concentrations in afforested soil (Tables 1 and 3) because they incorporate more soil carbon into biomass than bacteria^45^.

Soil microbial respiration is strongly dependent on soil C and N availability^53^,^54^. Higher basal microbial respiration in the afforested soils compared with cropland and uncultivated land (Fig. 3a) was possibly due to higher available C and N substrate (Table 1)^30^,^55^. Indeed, we found a strong correlation between the basal microbial respiration and soil C and N concentrations (Table 3). The basal microbial respiration increased in the afforested soils (Fig. 3a) could be also primarily attributed to the significant increase of total PLFA biomass in afforested land soils (Fig. 1c), because microbial communities are the participants of microbial respiration^6^. In contrast, the ratio of the basal microbial respiration to total PLFAs was lower in the afforested soils compared with uncultivated and cropland soils (Fig. 3b). This finding suggested that afforestation could lead to high microbial C utilization efficiency and decrease C loss on a per-unit-PLFA by respiration compared to uncultivated and cropland soils^55^, because higher F: B ratios in afforested soils than cropland soils (Fig. 1d) indicated fungi produce more biomass C per unit of C metabolized than bacteria, which would lead to greater C use efficiency^23^.

In summary, afforestation increased soil total PLFAs, various microbial types of PLFAs (i.e., bacterial PLFAs, fungal PLFAs, G^+^ bacterial PLFAs, G^−^ bacterial PLFAs, ACT PLFAs and AMF PLFAs) as well as soil basal microbial respiration. Variations in microbial types of PLFAs closely contacted with soil moisture, soil pH, soil temperature and soil C and N avallability. All of the environmental factors explained 86.5% of the variance of PLFAs, among which SON and LC were the crucial factors. However afforested soils decreased the basal microbial respiration on a per-unit-PLFA basis, suggesting that more carbon can be accumulated in afforested soils. Overall, shifts in microbial community structure caused by land use type conversion are very important for studying long-term C accumulation, soil restoration and reducing greenhouse gas in the future climate change scenarios.

## Materials and Methods

### Study area and experimental design

The experimental site is located in Wulongchi Experimental Station in the Danjiangkou Reservoir region (32°45 N, 111°13 E). The climate of this study area is a subtropical monsoon climate, with a mean annual temperature of 15.7 °C and monthly averages of 27.3 °C in July and 4.2 °C in January. The annual precipitation is 749.3 mm. The elevation of the site is approximately 280–400 m. The soil is a yellow brown soil (Chinese soil classification system) consisting of 11% sand, 41% silt, and 48% clay in the top 30 cm^39^. Human activities, such as deforestation and tillage, around the reservoir have caused soil erosion, water pollution and soil nutrient element losses in the region^39^. Approximately 18 years ago, large areas of cropland in this region were converted to woodland plantations of coniferous plants (*Platycladus orientalis (Linn.) Franco*)^39^ and shrubland plantations (*Sophora davidii (Franch.) Skeels*). Based on our surveys, farmers typically cultivated corn and rape in cropland. Corn and rape cultivation was managed by conventional agricultural practices including plowing to a 0.4 m depth, mineral fertilizations (approximately urea 375 kg ha^−1^ and urine ammonium 200 kg ha^−1^) and chemical weeding. The aboveground biomass of corn and rape was removed through harvesting.

Three sites of approximately 75 ha (500 m × 1500 m) were selected in September, 2014. The distances between the three sites were approximately 1 km. Four adjacent land types include woodland, shrubland, cropland and uncultivated land where no input of organic matter from trees and/or shrubs (i.e., the control) occurred at each site. A comprehensive survey of soil and vegetation was conducted in September 2014 to ensure the comparability (e.g., similar soil types and topographies) of the soil sampling plots among the four land types^37^,^48^.

### Field sample collection and measurements

In September, 2014, soils were sampled from each land type at each site. The 3 sub-plots (2 m × 2 m) were randomly set for each land use type. Soil from each subplot was sampled using a 5 cm diameter stainless steel soil cylinder. Samples were taken from two depths including 0–10 cm and 10–30 cm at three randomly selected locations within each sub-plot. A total of 18 soil samples (3 sub-plots in three sites with two soil layers) were collected to represent each land use type, with a grand total of 72 samples across all four land use types. Plant material and stones in the soil samples was manually removed with forceps. All fresh soil samples were sieved with a 2 mm sieve. Then, each fresh soil sample was divided into three subsamples. One subsample was air-dried for soil physicochemical analyses, one subsample was stored in a refrigerator at 4 °C prior to analysis for basal microbial respiration and one subsample was stored at −20 °C until Phospholipid Fatty Acids (PLFAs) analysis could be carried out.

Soil moisture was determined by oven-drying fresh soil at 105 °C to a constant weight. Soil pH was measured from soil water suspension (1:2.5 v: v) with a digital pH meter. The chemical fractionation of soil organic substrates was determined by the methods used by Rovira and Vallejo^56^. A portion of air dried soil (approximately 2000 mg) was treated with 5 mL 1 N HCl for 24 h to remove inorganic carbon for the measurements of soil organic carbon (SOC) and soil organic nitrogen (SON). Then, soil recalcitrant C (RC) and N (RN) were obtained by acid hydrolysis. Briefly, 500 mg samples were hydrolyzed with 20 mL of 5 N H_2_SO_4_ in sealed Pyrex tubes at 105 °C for 30 min. After cooling and being oven dried at 60 °C, the residue was hydrolyzed with 26 N 2 mL H_2_SO_4_ at room temperature overnight and then with 2 N H_2_SO_4_ at 105 °C for 3 h. After cooling, the unhydrolyzed residue was recovered by centrifugation and decantation of the supernatant liquid using deionized water to eliminate residual H_2_SO_4_. The residue was dried at 60 °C to a constant weight and analyzed for RC and RN by using an isotope ratio mass spectrometer (Thermo Finnigen, Delta- Plus, Flash, EA, 1112 Series, USA). Soil labile C (LC) is made by the difference between SOC and RC, similarly, soil labile N (LN) is made by the difference between SON and RN.

Soil microbial community structures were analyzed for PLFAs using the method described by Bossio and Scow^57^. Briefly, lipids were extracted from 8 g freeze dried soils in a 23 mL extraction mixture using chloroform: methanol: phosphate buffer (1:2:0.8 v/v/v). The extraction was transferred to a separatory funnel to separate overnight. Then phospholipids split into neutral, glyco- and phospho- lipids. To recover fatty acid methyl esters phospholipids were subjected to a mild-alkali methanolysis. Samples were then re-dissolved in hexane solvent containing nonadecanoic acid methyl ester (19:0) as an internal standard and were analyzed with an Agilent 6890 Gas Chromatograph equipped with an Ultra 2-methylpolysiloxane column. Bacterial fatty acid standards and MIDI eukaryotic method with Sherlock software (MIDI, Inc., Newark, DE) were used to identify peaks. Peaks were identified using bacterial fatty acid standards and MIDI peak identification software (MIDI, Inc., Newark, DE). We calculated the concentrations of each PLFA based on the 19:0 internal standard concentrations. Gram-positive bacteria (G^+^ bacteria) were identified by i14:0, i15:0, a15:0, i16:0, i17:0, a17:0^13^, gram-negative bacteria (G^−^ bacteria) were identified by 14:1 w5c, 16:1w9c, 16:1 w7c, cy17:0, 17:1 w8c, 18:1 w5c, 18:1w7c, cy19:0w8c^13^, ACT bacteria were identified by 10 Me 16:0, 10 Me 17:0, 10 Me 18:0 TBSA^13^, and total bacteria was the summary of 14:0, 16:0, 18:0, i14:0, i15:0, a15:0, i16:0, i17:0, a17:0, 14:1 w5c, 16:1w9c, 16:1 w7c, cy17:0, 17:1 w8c, 18:1 w5c, 18:1w7c, cy19:0w8c. Some other PLFAs were detected, which could also be considered as bacteria. However, we only used PLFA which have been completely identified and which were found in rather large amounts in all soil samples. The fungi were identified by 18:1 w9c and 18:2 w6,9c and 16:1 w5c, among them 16:1 w5c represented AMF fungi^47^,^55^. All of the PLFAs including above and15:1 w6c, 17:1 iso w5c, 20:4 w6,9,12,15c, 18:0 3 OH were considered to be representative of the total PLFAs of soil microbial community^13^,^55^,^58^. Each PLFA biomass and the sum of all PLFA biomass are expressed as μg PLFA g^−1^ dry soil.

The basal microbial respiration was determined by quantifying the carbon dioxide (CO_2_) released from 50 g dry-weight-equivalent fresh soil samples in a 500 mL glass jar during 30 days of incubation at 25 °C^59^. Briefly, 2-mm-sieved and root-picked fresh soil was added into a 500 ml Schott bottle, and then adjusted to 60% of the water-holding capacity (WHC). Empty 500 mL Schott bottles were used as blank control. All samples were pre-incubated at 25 °C for 7 days. Thereafter, water was added to the soil surface using a dropper to maintain 60% of soil WHC at this level throughout the experiment. 10 mL 0.5 mol L^−1^NaoH was added into small cups placed in the incubation bottles to absorb CO_2_, the NaOH solution was replaced after incubated 1, 4, 9, 16 and 23 days. The NaOH solution was titrated by 0.5 mol L^−1^ HCl soluiton to quantify the trapped CO_2_ [CO_2trapped_ (CO_2control_ is the quantity of trapped CO_2_ for blank control)] and their values were reported on a specific basis (i.e., per kg of soil). CO_2sample_ is the net emissions of CO_2_ for soil. It was calculated as follows:


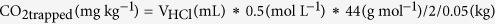



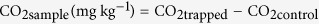


The basal microbial respiration was calculated by dividing sum of CO_2sample_ for 30 days (mg kg^−1^) with per unit time (i.e., hour)^59^. The basal microbial respiration to total PLFAs ratios were calculated by divided basal microbial respiration with total PLFAs^55^.

### Statistics

The data were examined for normality and log- or cubed root-converted to satisfy suppositions for statistical analysis. Two-way Analysis of Variance (ANOVA) was used to test the statistical significance of land use type, depths and their interactive effects on soil variables (soil moisture, temperature, pH, SOC, SON, C: N, LC, LN, RC, and RN), and soil microbial community structure (total PLFAs, bacterial and fungal PLFAs, F: B ratio, G^+^ bacteria PLFAs, G^−^ bacteria PLFAs, AMF PLFAs and ACT PLFAs). One-way ANOVA with Tukey’s HSD test was further used to test the statistical significance of land use type on soil basal respiration and soil microbial community structure of each soil layer. A paired t-test was further employed to compare the difference in soil microbial community structure, soil basal respiration rate and basal microbial respiration/total PLFAs ratios between the two soil layers at the same land use type. Pearson correlations analysis was performed among microbial communities, basal microbial respiration and soil properties across soil depth and land use types. Analysis for all of the data was carried out using SPSS 20.0 software. The Pearson correlation coefficients and redundancy analysis (RDA) were performed to quantify the correlations between soil microbial community and soil variables using Canoco5.0.

## Additional Information

**How to cite this article**: Zhang, Q. *et al*. Alterations in soil microbial community composition and biomass following agricultural land use change. *Sci. Rep.* **6**, 36587; doi: 10.1038/srep36587 (2016).

**Publisher’s note:** Springer Nature remains neutral with regard to jurisdictional claims in published maps and institutional affiliations.

## Figures and Tables

**Table 1:** Soil properties under different land use types at two soil depths.

Parameter	Depth (cm)	Land use type				F-value		
		W	S	C	U	D	L	D × L
Soil moisture	0–10	0.22 ± 0.02	0.18 ± 0.01	0.20 ± 0.04	0.12 ± 0.01	7.33*	22.37***	NS
	10–30	0.18 ± 0.01	0.14 ± 0.01	0.20 ± 0.01	0.12 ± 0.02			
Soil temperature		23.78 ± 1.11	25.18 ± 0.93	19.78 ± 0.17	26.85 ± 1.80	NS	40.10***	NS
pH	0–10	8.17 ± 0.08	8.17 ± 0.07	8.47 ± 0.12	8.53 ± 0.08	NS	21.82***	3.76*
	10–30	8.29 ± 0.03	8.34 ± 0.08	8.38 ± 0.07	8.53 ± 0.03			
SOC (g/kg)	0–10	18.81 ± 2.53	15.59 ± 2.37	4.14 ± 1.09	3.64 ± 0.56	13.60**	29.28***	6.11**
	10–30	7.26 ± 1.83	12.53 ± 0.61	3.18 ± 0.75	3.57 ± 0.17			
SON (g/kg)	0–10	1.30 ± 0.34	0.89 ± 0.06	0.58 ± 0.13	0.42 ± 0.03	23.45***	21.77***	5.66**
	10–30	0.69 ± 0.07	0.55 ± 0.03	0.48 ± 0.05	0.39 ± 0.05			
C:N ratio	0–10	14.27 ± 1.56	17.59 ± 3.40	7.15 ± 0.33	8.61 ± 0.91	NS	107.73***	9.47**
	10–30	10.48 ± 1.56	22.96 ± 0.52	6.65 ± 1.15	8.07 ± 2.28			
LC (g/kg)	0–10	5.40 ± 1.94	5.69 ± 0.87	0.68 ± 0.41	1.61 ± 0.67	17.58**	31.60***	5.97**
	10–30	1.73 ± 0.72	3.73 ± 0.09	0.27 ± 0.04	1.32 ± 0.12			
LN (g/kg)	0–10	0.67 ± 0.23	0.24 ± 0.15	0.18 ± 0.08	0.09 ± 0.04	5.03*	17.17***	3.03*
	10–30	0.34 ± 0.07	0.22 ± 0.04	0.11 ± 0.02	0.08 ± 0.07			
RC (g/kg)	0–10	13.41 ± 4.63	9.90 ± 1.50	3.46 ± 0.82	2.48 ± 0.17	10.79**	26.86***	6.03**
	10–30	5.53 ± 1.21	8.80 ± 0.09	2.91 ± 0.78	2.25 ± 0.09			
RN (g/kg)	0–10	0.62 ± 0.11	0.65 ± 0.15	0.40 ± 0.07	0.34 ± 0.07	23.66***	5.94**	5.57**
	10–30	0.35 ± 0.04	0.33 ± 0.04	0.37 ± 0.06	0.31 ± 0.03			

*Note*: W = woodland; S = shrubland; C = cropland; U = uncultivated land; D = depth; L = land use type. Values are mean (n = 9) with standard error. Statistical Significance of the effects of land use type, depth and their interactions on soil properties based on Two-way ANOVA (NS = not significant; *P < 0.05; **P < 0.01; ***P < 0.001; numbers are F-values).

**Table 2:** Statistical significance of the effects of land use type, depth and their interactions on soil microbial communities based on Two-way ANOVA (NS = not significant; *P < 0.05; **P < 0.01; ***P < 0.001; numbers are F-values).

	Depth (D)	Land use type (D)	L × D
Total PLFAs	53.96***	34.45***	7.56**
Bacterial PLFAs	65.02***	38.99***	12.60***
Fungal PLFAs	52.14***	40.33***	6.36**
F:B ratio	NS	23.48***	NS
G^+^ bacterial PLFAs	28.70***	23.16***	5.30**
G^−^ bacterial PLFAs	49.55***	25.54***	8.34**
AMF PLFAs	119.82***	33.07***	17.50***
ACT PLFAs	16.09**	16.68***	NS

**Table 3:** Pearson correlation coefficients (r) of microbial communities and basal microbial respiration on soil properties across soil depth and land use types (*P < 0.05; **P < 0.01; ***P < 0.001; numbers are F-values).

	Moisture	Temperature	pH	SOC	SON	C:N ratio	LC	LN	RC	RN
Total PLFAs	0.56**	0.13	−0.69**	0.91**	0.96**	0.46*	0.86**	0.85**	0.91**	0.80**
Bacterial PLFAs	0.57**	0.11	−0.70**	0.90**	0.96**	0.45*	0.85**	0.84**	0.90**	0.80**
Fungal PLFAs	0.44*	0.26	−0.71**	0.92**	0.91**	0.55*	0.90**	0.79**	0.91**	0.78**
F:B ratio	−0.48*	0.83**	−0.18	0.39	0.08	0.63**	0.49*	0.08	0.33	0.06
G^+^ bacterial PLFAs	0.62**	0.09	−0.67**	0.91**	0.98**	0.43*	0.84**	0.89**	0.92**	0.79**
G^−^ bacterial PLFAs	0.61**	0.06	−0.64**	0.88**	0.97**	0.38	0.81**	0.89**	0.89**	0.79**
AMF PLFAs	0.56**	0.03	−0.61**	0.79**	0.92**	0.28	0.73**	0.78**	0.80**	0.81**
ACT PLFAs	0.64**	0.04	−0.63**	0.89**	0.98**	0.41*	0.81**	0.91**	0.92**	0.76**
Basal microbial respiration	0.69**	−0.13	−0.56**	0.64**	0.90**	0.07	0.50**	0.92**	0.70**	0.58**
Basal microbial respiration/Total PLFAs	−0.70**	−0.46*	0.59**	−0.41*	−0.50*	−0.19	−0.28	−0.52**	−0.47*	−0.31
